# Calorie menu labeling on quick-service restaurant menus: an updated systematic review of the literature

**DOI:** 10.1186/1479-5868-8-135

**Published:** 2011-12-08

**Authors:** Jonas J Swartz, Danielle Braxton, Anthony J Viera

**Affiliations:** 1 Public Health Leadership Program, Gillings School of Global Public Health, University of North Carolina at Chapel Hill, Chapel Hill, NC, USA; 2 Nutrition Department, Gillings School of Global Public Health, University of North Carolina at Chapel Hill, Chapel Hill, NC, USA; 3 Department of Family Medicine, University of North Carolina at Chapel Hill School of Medicine, Chapel Hill, NC, USA

**Keywords:** Calorie label, menu label, nutrition information, restaurant label, Patient Protection and Affordable Care Act, obesity, food away from home, fast food

## Abstract

Nutrition labels are one strategy being used to combat the increasing prevalence of overweight and obesity in the United States. The Patient Protection and Affordable Care Act of 2010 mandates that calorie labels be added to menu boards of chain restaurants with 20 or more locations. This systematic review includes seven studies published since the last review on the topic in 2008. Authors searched for peer-reviewed studies using PUBMED and Google Scholar. Included studies used an experimental or quasi-experimental design comparing a calorie-labeled menu with a no-calorie menu and were conducted in laboratories, college cafeterias, and fast food restaurants. Two of the included studies were judged to be of good quality, and five of were judged to be of fair quality. Observational studies conducted in cities after implementation of calorie labeling were imprecise in their measure of the isolated effects of calorie labels. Experimental studies conducted in laboratory settings were difficult to generalize to real world behavior. Only two of the seven studies reported a statistically significant reduction in calories purchased among consumers using calorie-labeled menus. The current evidence suggests that calorie labeling does not have the intended effect of decreasing calorie purchasing or consumption.

## Introduction

As part of the Patient Protection and Affordable Care Act of 2010, lawmakers passed a requirement that all chain restaurants with 20 or more locations include calorie information on all menus. If enacted, the policy will require these restaurants to list at the very least the calorie information in the foods and beverages they serve. This new legislation builds upon efforts already underway in some states to provide consumers with more information about the foods they purchase away from the home [1].

Menu labeling is one of many policy approaches that has been proposed to address the increasing prevalence of overweight and obesity in the United States [2,3]. In particular, researchers and policymakers have begun to focus on how the increasing reliance on food away from home in the US diet [4,5] may be contributing to poor health [6,7]. Food away from home now accounts for over 30% of daily caloric intake and 50% of yearly food spending [4-6]. This trend is concerning because foods consumed away from home typically have more calories, fat and sodium than foods prepared in the home [4]. Frequent consumption of food away from home has also been linked to higher rates of overweight and obesity [8,9].

In an effort to address the role of food away from home in the overweight and obesity epidemic, several states, cities and counties have passed menu labeling laws starting with New York City in 2006. The New York City law required restaurants with 15 or more locations to list calorie information for each item on the menu in a prominent location both on menu boards and menus and began enforcement in 2008. Shortly thereafter, in 2010, Congress passed the Patient Protection and Affordable Care Act which included a national menu labeling law for all restaurants with 20 or more locations [1,10].

Though momentum has gathered behind menu labeling policies as a tool for combatting overweight and obesity, evidence to support its efficacy is less robust. In a 2008 review, Harnack and French were able to identify only six studies that tested the effects of calorie labeling on consumer choice [5]. They concluded that from the current evidence, the effects of calorie labeling appeared to be weak or inconsistent [5]. However, they also noted major methodological flaws in each of the studies [5].

In this systematic review, we update Harnack and French's findings with more recent evidence. The purpose of this paper is to use current literature to answer the question of whether calorie labeling on menus at restaurants and cafeterias has an effect on consumer purchasing and eating behaviors.

## Methods

### Search strategy

The most recent review of the literature was published in 2008 and included articles published through 2006. In the current search, conducted in August 2011, we sought studies with publication dates from 2006-August 2011. We used PUBMED and Google Scholar World Wide Web search engines to identify relevant studies. Initial PUBMED searches with MeSH terms including "food labeling", "fast foods" and "choice behavior" yielded few results. We therefore broadened our search to include the following keywords: "calorie labeling", "menu labeling" and "point-of-purchase labeling". We supplemented our findings with hand searches from the reference lists of articles and reviews [7,11-15].

### Article selection

To be included, studies must have used an experimental or quasi-experimental design comparing a calorie-labeled menu with a no-calorie menu. This review includes studies conducted in laboratories, college cafeterias, and fast food restaurants. Only studies that measured purchasing behavior or consumption of ready-to-eat meals were included. Our search was restricted to English-language in peer-reviewed publications.

### Data extraction

One author (JS) extracted standardized information including study aims, study type, sample population, and outcomes in a spreadsheet to facilitate comparison and synthesis. The table also included information about methodological strengths and weaknesses of the studies.

### Quality assessment

Quality was graded with the assistance of an instrument developed by the investigators based on standard critical appraisal criteria. The instrument required assessment of a variety of study procedures including study design, randomization, blinding, minimization of selection bias, minimization of measurement bias, and minimization of confounding bias. For each applicable variable, two authors (JS and AV) independently assessed each article and assigned scores of 2 for good, 1 for fair and 0 for poor. To achieve a quality rating of good, studies had to have an average score greater than or equal to 1.5 and could not receive scores of 0 for any individual variable. An average score less than or equal to 0.5 was considered to indicate a study with poor quality. Studies with scores in between were considered fair quality studies. We excluded the randomization category for observational studies. After independently scoring each article, the two raters conferred to discuss any discrepancy in overall quality rating.

## Results

### Search results

**Figure 1:**
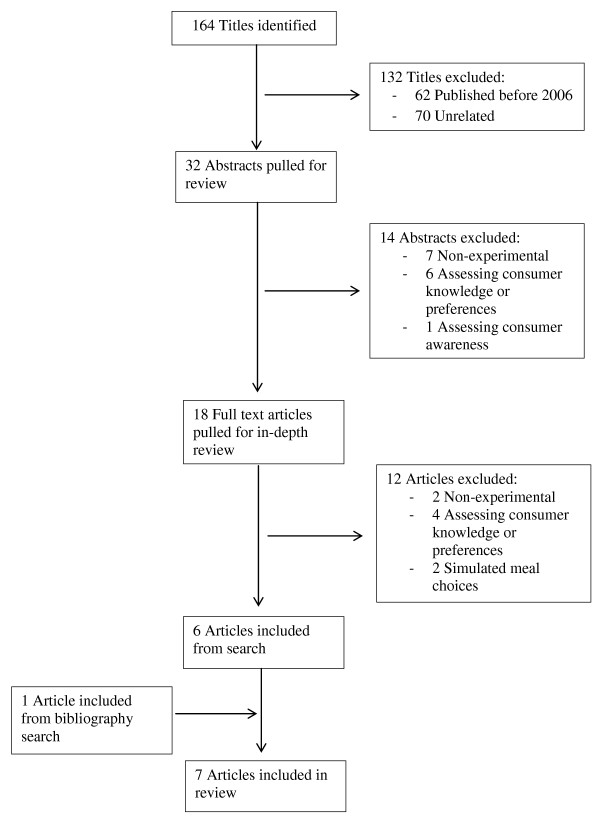
**Search and selection flow chart**.

The initial search produced 164 citations on PUBMED. One author (JS) screened titles for relevance and further examined 32 abstracts. Eighteen articles underwent full text review, after which 12 articles were excluded. One article was included from a hand search. Seven articles met the inclusion and exclusion criteria described above (Figure 1).

### Study design

Included articles reported on studies conducted in two different types of settings; five articles reported on natural experiments of calorie menu label implementation in real world settings [6,16-19] and two involved researcher manipulated variables in laboratory settings [20,21] (Table 1).

**Table 1:** Characteristics of included studies

Reference	Design and Presence of Comparison Group	Intervention/Measures	Setting	Number of Subjects/Restaurants	Result
**Real world setting**					

Elbel et al. (2011) [17]	Natural experiment, pre/post intervention comparison and with matched community	Calorie labels added to chain restaurant labels in New York City. Survey administered outside fast food restaurants.	New York City and Newark, NJ (as comparator). Fast food restaurants in low-income neighborhoods	349 children and adolescents	Mean calories purchased in NYC pre and post labeling 643 v 652 (p = 0.82), Newark 611 v 673 (p = 0.37).

Elbel et al. (2009) [18]	Natural experiment, pre/post intervention comparison and with matched community	Calorie labels added to chain restaurant labels in New York City. Survey administered outside fast food restaurants.	New York City and Newark, NJ (as comparator). Fast food restaurants in low-income neighborhoods	1156 adults over 18	Regression-Adjusted nutrient content in NYC and Newark before and after with 95% CI. NYC: 825 (779, 870) post 846 (758, 889). Newark 823 (802, 890) post 826 (746, 906).

Finkelstein et al. (2011) [19]	Natural experiment, pre/post intervention comparison with matched communities	Calorie labels added to chain restaurant labels in King County, WA, then drive-thru lanes. Total monthly transactions and calories per transaction.	King County, WA and several stores from surrounding area	21 randomly selected Taco Time locations and 7 locations outside King County	Calories per transaction King County pre-period: 1,211 v post-period 1: 1,217 v post-period 2: 1,214. Calories per transaction Control pre-period: 1,391 v. post-period 1: 1,392 v post-period 2: 1,376.

Chu et al. (2009) [6]	Quasi-experimental, single group interrupted time series	Calorie labels added to entrees in college dining hall. Used electronic sales data to track calories of entrees sold.	Dining hall, Ohio State University	NA	Calories per entrée sold at pre 645.5, First day of tx period -12.4 (p = 0.007), decreased of 0.298 calories/day), post treatment increases 1.512/day

Dumanovsky et al. (2011) [16]	Cross sectional surveys pre/post calorie menu label implementation	Calorie labels added to chain restaurant labels in New York City. Survey administered outside fast food restaurants.	New York City fast food chains	7309 adult customers in 2007 and 8489 in 2009, 168 locations of 11 fast food chains	No change in mean calories purchased overall chains from 2007 to 2009, 828 v 846 kcal (p = 0.22). Three chains show reduction in mean calories per purchase: McDonalds (829 v 786, p < 0.02), Au Bon Pain (555 v 475, p < 0.001), KFC (927 v 882 kcal, p < 0.001). One chain significant increase: Subway (749 v 882, p < 0.001).

**Laboratory setting**					

Harnack et al. (2008) [20]	Non-blinded randomized controlled trial	Order from 4 menu labeling conditions, control that lists items with standard pricing, Item + Calorie menu, Item + Non-value menu pricing, Calorie + Non-Value menu pricing. Measured calories ordered and calories consumed	Conference room of suburban hotel and church basement in Minneapolis St. Paul, MN	594 adolescents and adults 16 or older	Mean calories ordered: Calorie 873.6, Price 881.7, Calorie+Price 842.3, Control 827.5 (p = 0.62); Mean calories consumed: Calorie 804.7 Price 813.3 Calorie+Price 761.0 Control 739.0 (p = 0.25)

Roberto et al. (2010) [21]	Non-blinded randomized controlled trial	Participants order from 3 menu labeling conditions, one that lists the items, one that lists items and calories, one that lists items, calories and daily guideline calories. Measured calories ordered and calories consumed	Laboratory in New Haven, CT	303 adults 18 and older	Mean calories ordered: Control 2189, label condition 1862 (p = 0.03), label + info condition (1860, p = 0.03), no significant difference between two label conditions. No significant difference in calories consumed overall (p = 0.12).

### Study quality

All studies included in this review had methodological shortcomings. Despite these limitations, two studies were judged by the two raters to be of good quality [19,20] and five to be of fair quality [6,16-18,21] (Table 2).

**Table 2:** Quality assessment of included studies

Reference	Study design*	Randomization	Blinding	Selection bias	Measurement bias	Confounding	Overall quality based on score average**
**Real world settings**							

Elbel et al. (2011) [17]	1	NA	0	1	2	0	Fair

Elbel et al. (2009) [18]	1	NA	0	1	2	0	Fair

Finkelstein et al. (2011)[19]	1	NA	2	2	2	1	Good

Chu et al. (2009) [6]	0	NA	2	2	2	1	Fair

Dumanovsky et al. (2011) [16]	0	NA	0	1	2	0	Fair

**Laboratory settings**							

Harnack et al. (2008) [20]	2	2	1	2	2	1	Good

Roberto et al. (2010) [21]	2	2	0	2	2	1	Fair

NA, not applicable

* Study design: Randomized controlled trials received a score of 2, cohort studies with comparison groups received a score of 1, and single group studies (with no comparison group) received a score of 0

**Quality scoring: 2 for good (or low potential for bias), 1 for fair (or moderate potential for bias), 0 for poor (or high potential for bias). To achieve a final quality rating of good, studies had to have an average score greater than or equal to 1.5 and could not receive scores of 0 for any individual variable. An average score less than or equal to 0.5 would have been considered to be a study with poor quality. Scores in between were considered fair quality studies.

### The effect of calorie menu boards on calorie ordering and purchasing

All seven studies compared calorie ordering and purchasing in two conditions: calorie label versus no calorie label. Two studies reported that calorie menu labels reduced the calories purchased [6,21], one reported significant reductions in calories purchased at some chains (but not others)[16], three reported no effect on calories purchased [17,18,20] and one reported a slight increase in calories purchased [19].

Among the observational studies, Elbel et al. found that in New York City, purchasing behavior of children and adolescents did not differ before and after calorie labels were implemented on menu boards, with patrons purchasing a mean of 643 calories before labeling and 652 calories (p = 0.82) after restaurants introduced menu labels [17]. The authors also observed a non-significant change in purchasing behavior over the same time period among children and adolescents in Newark, NJ, where calorie labels were not introduced (611 vs. 673 calories, p = 0.37)[17]. A companion study of adults also showed a non-significant difference in New York City [18]. Adults purchased a regression-adjusted mean of 825 calories (95% CI: 779-870) before calorie labeling and 846 calories (95% CI: 758-889) after calorie labeling. There was also a non-significant trend among adults in Newark, NJ with 823 calories (95% CI: 802-890) in the pre-labeling time period and 826 calories (95% CI: 746-906) in the post-labeling time period [18].

Also in New York City, Dumanovsky et al. collected survey and purchase data before calorie labeling implementation in 2007, and nine months after implementation in 2009. They collected data from the 11 largest fast food chains, and found no change in mean calories purchased overall between study periods in 2007 and 2009 (828 vs. 846 calories, P = 0.22)[16]. When examining data for each chain individually, they found a reduction in mean calories purchased for three chains (McDonald's 829 vs. 785 calories, P = 0.02; Au Bon Pain 555 vs. 475 calories, P < 0.001; KFC 927 vs. 868 calories, P < 0.01), no significant difference for 7 chains (Burger King, Wendy's, Popeye's, Domino's, Pizza Hut, Papa John's, Taco Bell), and an increase for one chain (Subway 749 vs. 882, P < 0.001)[16]. The study did not include a control population.

Though it was a small change, Finkelstein et al. did observe a small, statistically significant (but we do not think clinically significant) increase in calories purchased per transaction after calorie labels were added to menus in King County, WA [19]. Patrons purchased 5.7 (p < 0.05) more calories after calorie labels were introduced on menu boards inside restaurants, and 2.9 (p < 0.05) more calories after calorie labels were introduced on drive-thru menu boards. In the control county, they did not observe a significant trend. Moreover, a difference-in-difference regression analysis found that calories per transaction were not reduced after the legislation [19].

In a study of entrée purchasing in a college dining hall, Chu et al. reported a significant but modest decrease in calories per entrée sold during the two weeks that calorie labels were posted on menu boards (treatment)[6]. They calculated average calories per sale using sales data furnished by the cafeteria. In the two weeks before posting calorie information (pretreatment), the average energy content was 646.5 calories per entrée. This average dropped 12.4 calories per entrée sold on the first day of calorie posting (p = 0.007) and remained lower throughout the treatment period. Though statistically significant, an average reduction of 12.4 calories may not be clinically significant.

In contrast to studies utilizing only purchasing behavior, the two experimental studies conducted in laboratory settings allowed researchers to measure both calories ordered and calories consumed (discussed below)[20,21]. Harnack et al. found no significant difference in calories ordered among four menu labeling conditions manipulating availability of calorie labels and value pricing (calorie labels + value pricing 874, calorie labels without value pricing 842, no calorie labels + no value pricing 882, and no calorie labels + value pricing (control) 828 calories, p = 0.62)[20].

Roberto et al. tested three types of menus: one had no calorie labels (no label), one had calorie labels (calorie), and one had calorie labels and a statement that the recommended daily caloric intake was 2000 calories (calorie + information)[21]. They found that menu type had a statistically significant effect on calorie ordering (p = 0.04). Significant differences were found between the no label and calorie labeled menus (no label 2189, calorie 1862 calories, p = 0.03), and also a significant difference between the no label menus and the calorie + information menus (1860 calories, p = 0.03). The difference between the calorie menus and calorie + information menus was not statistically significant (p = 0.99). It is not clear why the difference in calories ordered between the groups appears to be more clinically significant than those noted in other studies [21]. However, the average number of calories ordered was also high compared to previous studies, which may account for some of this difference.

### The effect of calorie menu labels on calorie consumption

As noted above, two studies measured calories consumed in addition to calories ordered or calories purchased [20,21]. The distinction is an important one since consumers might theoretically respond to calorie posting on menus by changing the amount they eat rather than the amount they order. Harnack et al. found, however, that participants overall did not differ significantly in the number of calories they consumed by menu type (no label 739, calorie labels 805, no value pricing 761 calories, p = 0.25)[20]. Subgroup analysis did demonstrate a difference in calories consumed. Men in groups with menus listing calorie information and those without value pricing consumed more calories than those with control menus (p = 0.01)[20].

Roberto et al. also found no significant difference between calorie consumption when they examined consumption by menu type overall (no label 1459 vs. calorie label 1335 vs. calorie + information 1256, p = 0.12)[21]. However, when they combined the two calorie label menus and compared them to the no label menu, they did find those in the labeled condition consumed fewer calories than those in the no label condition (label 1286 vs. no label 1466, p = 0.04)[21]. The credibility of this result is questionable considering the exploratory circumstances in which it was found. The average number of calories consumed was very high for a single meal.

### Sales volume

Two studies reported measures of sales volume, neither of which found a significant difference in sales volume between periods with and without calorie posting [6,19]. Finkelstein et al. found no significant differences in the rate of ordering healthy or unhealthy menu items before and after calorie posting [19]. Chu et al. reported a significant decrease in the sale of entrées with the highest energy content during the treatment period (slope = -0.766 entrees/day, p = 0.007) and an increase in sale of entrées with the highest energy content after the treatment period (slope = 1.541 entrees/day, p = 0.005)[6].

## Discussion

Overall, the studies included in this review suggest that in both real world and experimental settings, calorie menu labeling has no effect or only a modest effect on calorie ordering and consumption. These results do not provide strong support for arguments that national expansion of calorie menu labeling will reduce rates of overweight and obesity. This evidence update supports the findings of the previous review from 2008 [5]. However, we should consider limitations of the current evidence as well as other important caveats before judging these policies.

### Strengths and limitations of current evidence

As noted above each study included in this review had methodological limitations. Finkelstein et al. provides the best evidence regarding implementation of calorie menu labeling in a real world setting and was the study with the highest quality overall included in this review. The researchers analyzed complete sales data furnished by a chain of restaurants in and surrounding King County, WA in a 13-month period during which the county implemented a calorie menu labeling requirement. The study provided more compelling data than three studies conducted in New York City, largely because researchers were able to track total monthly transactions and had complete sales data. In New York City, although researchers gathered data from multiple restaurants, they had no measure of overall volume of sales. This is important because one possible effect of calorie menu labeling is that consumers choose not to patronize restaurants where unhealthy choices dominate.

With the observational studies it is entirely possible that confounding factors were responsible for the reported effects of calorie labeling. In New York City and King County, WA, researchers did not measure consumption patterns which could have changed with calorie labeling even if ordering patterns remained constant. Moreover, none of the observational studies could account for environmental factors, such as public education campaigns accompanying the policy implementation, which might have contributed to behavior change over the course of the study. Since four of five studies showed that calorie labels did not lead to decreased calorie ordering, we can feel comfortable that results are not skewed toward a positive result.

Although randomized trials are considered stronger designs because they have the potential to minimize confounding and selection bias, the two trials included in this review cannot easily be generalized to real world behavior and do not necessarily provide more compelling data than the observational research. Regardless of efforts to improve real world applicability or conceal study aims, participants are likely to order and eat differently when they are being monitored and eating in groups. Moreover, in the real world, people have the choice to forgo quick-service restaurant meals in favor of those prepared at home (though we are doing so less frequently as a nation)[4,15].

The two experimental studies included in this review reported conflicting results on calorie ordering and consumption, which could be a product of study design, demographic variables, label design or measurement. Participants in the Roberto et al. research [21], which demonstrated the largest effect from calorie labeling, ordered over twice as many calories and consumed several hundred more calories than those in any other study [21]. The authors offer no explanation for the increased caloric consumption, but it is possible that ordering and eating habits deviated from the norm due to the nature of the eating environment.

### Directions for future research

Current research on calorie menu labeling suffers from two basic deficiencies. Observational studies are insufficient for drawing causal inferences and experimental studies conducted in laboratory settings cannot simulate real world behavior, particularly when repeated exposure to menu labels may be required to influence choice [5,14,15,20]. With industry and governmental participation, large scale randomized trials could be conducted by gradually staging calorie menu label implementation on a state or county level in a randomized fashion. Researchers could then monitor ordering patterns, sales volume and even trends in overweight and obesity during parallel time periods in areas with and without labeling [15]. These large trials would provide the strongest evidence were researchers able to measure the number of calories consumed, not just the number ordered, as some consumers may respond to calorie labeling by changing their eating and not their ordering habits. However, both funding and lack of industry cooperation would likely constitute significant barriers to this sort of research [19,20].

Several of the studies in this review indicated that not all consumers or participants were aware of calorie labels [17,18,20]. Accompanying labels with educational materials to increase awareness and explain their use improves effects on food choice [5]. Future research should examine whether such efforts could also improve efficacy of calorie menu labeling. Researchers should also consider whether health and lifestyle variables--dieters vs. non-dieters, for example--affect how labeling influences choice.

In addition to how calorie labels affect consumers, future research should focus on the behavior of the food service industry. None of the studies monitored industry response to calorie menu labeling, including altering menus to improve nutrition profiles of current offerings or add healthier items. Unfortunately, because of low health literacy, lack of understanding of nutrition labels and misestimation of nutritional content in restaurant meals [4,14], consumers may also be susceptible to deliberate or inadvertent manipulation by the restaurant industry. Stated caloric estimates may also be incorrect [22]. As menu labeling becomes more prominent nationally, policy makers and industry regulators must be attentive to the truthfulness and clarity of new labels. Future research should also explore understanding of various label formats to make sure that responses correspond to intended meaning.

### Limitations of the current review

This review was limited in scope, in particular by restricting eligibility to studies published after 2006. In their previous review, Harnack and French concluded that calorie labeling might have a small effect on choice behavior [5]. The search also did not include other sources of peer reviewed or grey literature (a recent issue brief sites several unpublished studies of calorie menu labels)[15]. Finally, several excluded studies published since 2006 measure behavioral intent using calorie menu labels. That literature may provide an additional perspective on the potential for calorie menu labels to influence food choices.

## Conclusion

From the evidence included in this review, it appears that calorie menu labeling does not have the intended effect of decreasing calorie ordering and consumption from quick-service restaurants. We also need longer-term, scientifically rigorous studies to determine whether prolonged exposure to calorie labels has an effect on rates of overweight or obesity, the health outcome of interest [15].

In the meantime, we must proceed with caution in widespread implementation of an unproven policy with social and monetary costs, especially since the effort may detract attention from other effective strategies to combat overweight and obesity or have inadvertent effects [15]. Given that a majority of US consumers indicate that they want calorie menu labeling [14,23], and the policy now seems imminent, knowledge of successful strategies as well as potential negative ramifications should be carefully considered when deciding how the policy will be operationalized and implemented.

## Competing interests

The authors declare that they have no competing interests.

## Authors' contributions

JS conducted the literature search, reviewed each of the studies and drafted the manuscript. AV reviewed the studies and made contributions to multiple revisions of the manuscript. DB provided guidance about the content of the review, suggested pertinent literature, and contributed to multiple revisions of the manuscript. All authors read and approved the final manuscript.

## Authors' information

Contact information:

Danielle Braxton, MPH RD LDN

UNC Gillings School of Global Public Health

2200 McGavran-Greenberg Hall, CB #7461

Chapel Hill, NC 27599-7461

furci@unc.edu

Anthony J. Viera, MD MPH

UNC-Chapel Hill, DOFM

CB# 7595, 590 Manning Dr.

Chapel Hill, NC 27599-7595

anthony_viera@med.unc.edu
